# Maternal Gastrointestinal Nematode Infection Up-regulates Expression of Genes Associated with Long-Term Potentiation in Perinatal Brains of Uninfected Developing Pups

**DOI:** 10.1038/s41598-019-40729-w

**Published:** 2019-03-12

**Authors:** Manjurul Haque, Kristine G. Koski, Marilyn E. Scott

**Affiliations:** 1 Institute of Parasitology, McGill University (Macdonald Campus), 21 111 Lakeshore Road, Ste-Anne-de-Bellevue, Québec H9X 3V9 Canada; 2 School of Human Nutrition, McGill University (Macdonald Campus), 21 111 Lakeshore Road, Ste-Anne-de-Bellevue, Québec H9X 3V9 Canada

## Abstract

Establishment of neural networks critical for memory and cognition begins during the perinatal period but studies on the impact of maternal infection are limited. Using a nematode parasite that remains in the maternal intestine, we tested our hypothesis that maternal infection during pregnancy and early lactation would alter perinatal brain gene expression, and that the anti-inflammatory nature of this parasite would promote synaptic plasticity and long-term potentiation. Brain gene expression was largely unaffected two days after birth, but in seven-day old pups, long-term potentiation and four related pathways essential for the development of synaptic plasticity, cognition and memory were up-regulated in pups of infected dams. Interestingly, our data suggest that a lowering of Th1 inflammatory processes may underscore the apparent beneficial impact of maternal intestinal infection on long-term potentiation.

## Introduction

**Figure 1:**
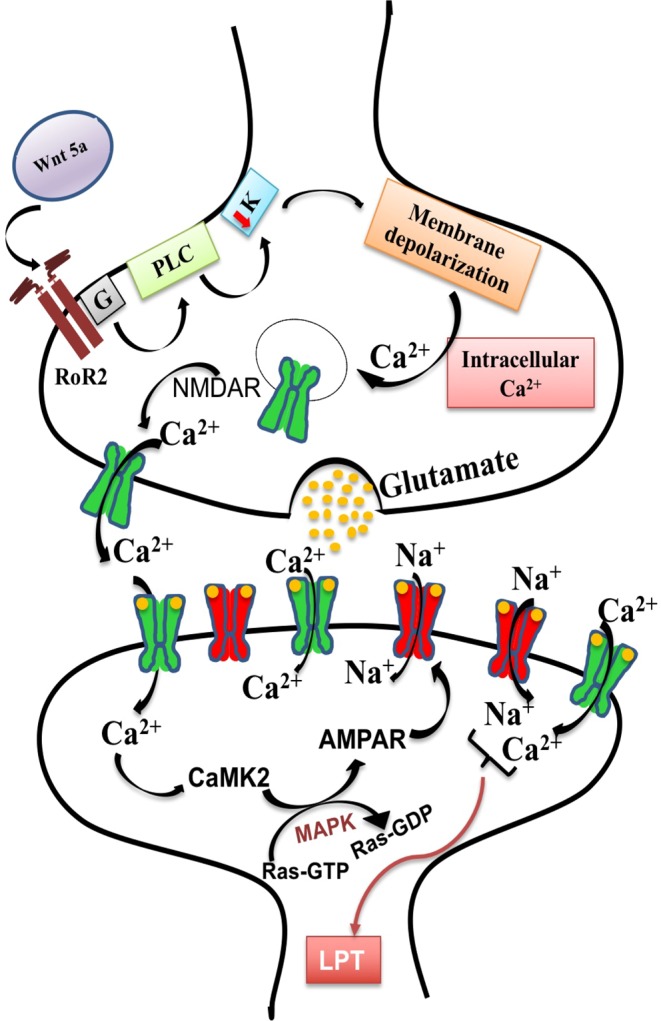
Schematic of pathways associated with long-term potentiation in the pre-and post-synaptic membrane. Binding of Wnt5a to surface receptors on the pre-synaptic cell membrane (initiation of the Wnt signaling pathway) leads to mobilization of Ca2+ from intracellular stores (initiation of Ca2+ signaling pathway) and expression of N-methyl-D-aspartic acid receptors (NMDARs) on both pre-synaptic and postsynaptic membranes (initiation of the glutamatergic synapse pathway)^3^. Expression of NMDARs on the post-synaptic membrane facilitates the entry of Ca2+ which in turn activates calcium-calmodulin dependent protein kinase II (CaMKII) and mitogen-activated protein kinases (MAPKs) (initiation of the MAPK signaling pathway)^34^. Together NMDARs and MAPK lead to phosphorylation and translocation of α-amino-3-hydrozy-5-methylisoxazole-4-propionic acid receptors (AMPARs) to the post synaptic membrane^36^ where they increase synaptic conductance leading to long- term potentiation.

Processes that underscore cognition and memory begin early in development through induction of five key neurological pathways that prime long-lasting plasticity in synaptic strength^1–3^. This phenomenon is referred to as long-term potentiation and relies on early, efficient transport of calcium and glutamate across the glutamatergic synapse. A key early step is the transition from “silent” glutamatergic synapses where only a few glutamate receptors are present on the post-synaptic membrane to the persistent activation of the N-methyl-D-aspartic acid (NMDA) and α-amino-3-hydrozy-5-methylisoxazole-4-propionic acid (AMPA) receptors for calcium and glutamate^4^. This relies on up-regulation of Wnt, glutamatergic, calcium and MAPK signaling pathways as well as the long-term potentiation pathway (Fig. 1)^1–3^. When these five pathways are established during early brain development, they are more likely to become “hard-wired” leading to long-term synaptic plasticity that enhances cognition and memory^5^. In mice, synaptic connections begin to form just before birth and most neurons have established synaptic connections within a week of birth^6^. Thus, the early postpartum period provides a developmental window where maternal factors may not only influence pup brain gene expression but also influence long-term potentiation.

Maternal factors including *in utero* exposure to glucocorticoids^6^ and toxins^7^, neonatal separation^8^, grooming and licking of pups^9^ and immune activation^10^ have been shown to impair development of long-term potentiation during the perinatal period. In addition, systemic and intrauterine viral and bacterial infections that induce strong pro-inflammatory (Th1) responses during pregnancy^11^ have been linked to perinatal brain damage in response to resulting immune activation and neuro-inflammation^12^. Also, intrauterine infection with *Escherichia coli* and the associated increase in the pro-inflammatory cytokine, IL1-β, has been associated with a reduction long-term potentiation and impaired responses to motor and cognitive tests^13^. In contrast, we recently explored the impact of low dose infection of pregnant mice with a nematode that is restricted to the maternal intestine and that induces anti-inflammatory Th2 responses^14^. We observed up-regulated expression of genes related to neurodevelopment and synaptic plasticity in response to maternal infection, in the fetal brain at embryonic day 18. Together, these studies show that maternal conditions that induce Th1 responses may have harmful effects on perinatal brain development but that conditions that promote Th2 responses may have beneficial effects.

The present study was conducted using the mouse intestinal nematode, *Heligmosomoides bakeri*, a common model for the intestinal nematodes that infect over 24% of the world’s population^15^. After infective larvae are ingested, the parasite remains in the intestine of the host, first in the submucosa as a larval stage, and then in the lumen of the small intestine where adult worms mate and females release eggs in the faeces^16^. The strong anti-inflammatory nature of this parasite is evident in Th2 responses both in the local tissue and in circulation of infected mice^17^, and in the enhancement of the Th2 response over that normally observed in pregnancy^18^. Furthermore, this infection reduces symptoms of auto-immune diseases that induce a strong inflammatory response^16^. Thus, this model is suitable for testing the intriguing possibility that an infection that remains restricted to the maternal intestine and that is known to induce a Th2 response may have a positive impact on pathways associated with long-term potentiation (Fig. 1) in the uninfected pups.

The study had three objectives. First, we documented differential brain gene expression at postnatal day 2 (P2) and P7 and compared it with the data from embryonic day 18 (E18) from our previous experiment^14^, in order to record the impact of maternal infection on the developmental progression of brain gene expression in the uninfected pups. Second, we used gene ontology and pathway analyses to determine whether maternal nematode infection altered expression of individual genes and pathways associated with long-term potentiation in the post-partum brain. Finally, we used pathway analysis and interrogated the gene expression database to determine if maternal infection altered neuro-inflammation or the cytokine environment in the pup brain on P7.

## Results

### Maternal nematode infection changed developmental progression of brain gene expression

**Figure 2:**
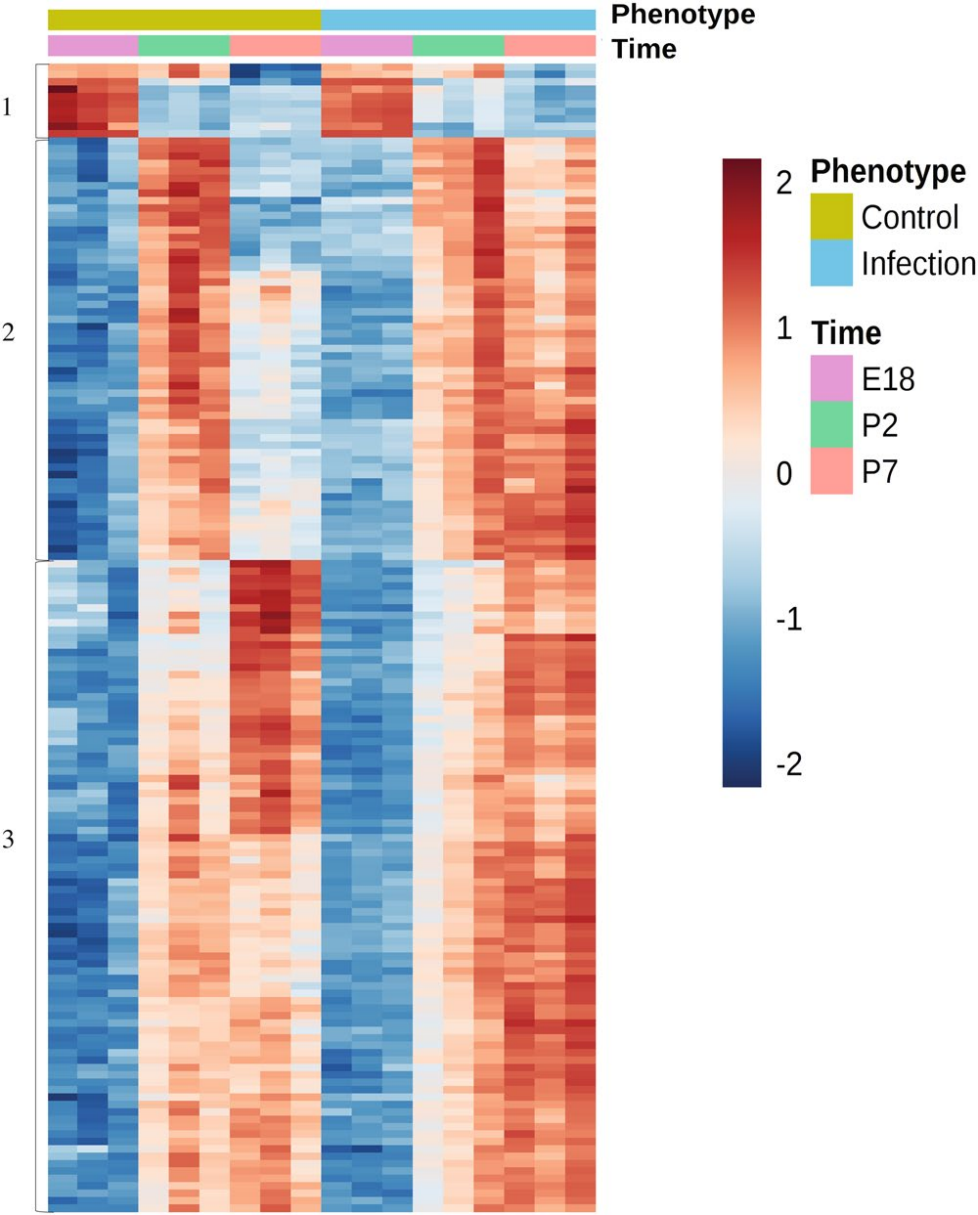
Heatmap generated after elimination of non-significant genes by ANOVA2 with Bonferroni multiple testing revealing 3 temporal patterns of differential gene expression between control and infection groups at embryonic day 18 (E18), postnatal day 2 (P2) and postnatal day 7 (P7). Pattern 1 gene expression was higher on E18 than P2 and P7 in both control and infected groups. Expression of pattern 2 genes increased from E18 to P2 in both control and infected groups and highly expressed at P7 in the infected group but not the control group. Expression of genes in patterns 3 progressively increased from E18 to P2 to P7 irrespective of the infection status of the dams.

Time series analysis provided a heatmap of the full developmental profile of brain gene expression from E18 to P2 to P7 in control and infected groups (Supplementary Fig. 1). When only the differentially expressed genes were visualized, three developmental patterns emerged (Fig. 2). In pattern 1, gene expression was higher on E18 than on P2 and P7 in both control and infected groups, reflecting developmental down-regulation of pattern 1 genes post-partum. In pattern 2, gene expression increased from E18 to P2 in both control and infected groups but remained elevated at P7 only in the infected group. In pattern 3, gene expression increased from E18 to P2 to P7 irrespective of the infection status of the dams. Expression of the 100 most highly ranked genes in the time series analysis differed between the infected and control groups only on P7 and was up-regulated in 90 of these 100 genes (Supplementary Dataset 2), a pattern consistent with pattern 2 genes (Fig. 2).

Analysis of the P2 and P7 datasets revealed that only three genes were differentially expressed in the P2 pup brain: one gene (high mobility group AT-hook 1B [*Hmga1b*]) was up-regulated and two genes (MORN repeat containing 2 [*Morn2*] and Gm38402 predicted gene [*Gm38402*]) were down-regulated. In contrast, at P7, 2751 brain genes were up-regulated and 2985 were down-regulated in the infected compared with the control group (Supplementary Dataset 1), and the majority had a log2 fold change between 1 and 2 (Supplementary Fig. 2). Several genes known to be critical for postnatal brain development were up-regulated on P7 including thrombospondin 1 (*Tsp1*) which promotes synaptogenesis^19^, orthodenticle homeobox 1 (*Otx1*) which is essential for dendritic growth of neurons^20^ and bromodomain PHD finger transcription factor (*Bptf*) which helps in neurodevelopment^21^. In addition, expression of transcription factors, receptors for glutamate, GABA, glycine, cholinergic, dopamine and serotonin, several ion channels (calcium, potassium, sodium, chloride, and potassium/chloride co-transporter), synaptic proteins, cell adhesion molecules, and cell signalling pathways (Wnt, Bmps and hedgehogs) was up-regulated in the infected group at P7 (Supplementary Dataset 3).

### Maternal nematode infection altered P7 brain gene ontologies, pathways and protein-protein interaction networks

#### Gene ontology analysis

**Figure 3:**
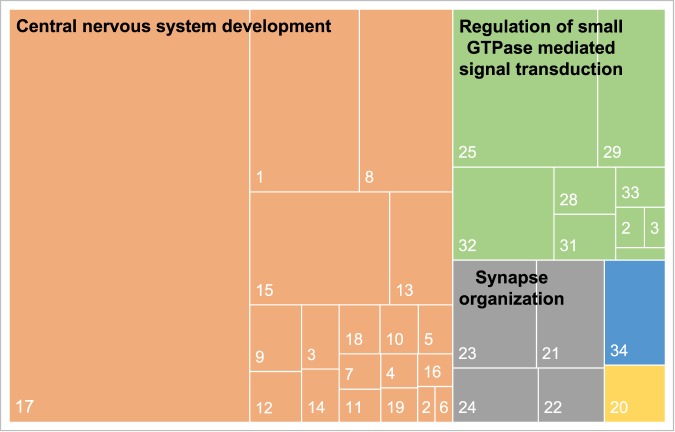
TreeMap visualization of biological process GO terms (small rectangles) up-regulated. In REVIGO. Superclusters of loosely related terms are visualized using different colors. The size of each rectangle reflects the p-value of the GO term. Where 1. Central nervous system development; 2. Regulation of neuron migration; 3. Neuromuscular process; 4. Neuromuscular process controlling balance; 5. Nerve development; 6. Embryonic camera-type eye morphogenesis; 7. Glomerulus development; 8. Post-embryonic development; 9. Gland morphogenesis; 10. Artery development; 11. Neuroepithelial cell differentiation; 12. Embryonic pattern specification; 13. Developmental growth involved in morphogenesis; 14. Neural tube closure; 15. Head development; 16. Cranial nerve development; 17. Cell morphogenesis; 18. Peripheral nervous system development; 19. Autonomic nervous system development; 20. Neural precursor cell proliferation; 21. Synapse organization; 22. Regulation of synapse structure or activity; 23. Plasma membrane organization; 24. Regulation of cell size; 25. Regulation of small GTPase mediated signal transduction; 26. Positive regulation of JUN kinase activity; 27. Negative chemotaxis; 28.Posttranscriptional gene silencing by RNA; 29. Steroid hormone mediated signaling pathway; 30. Regulation of glutamate receptor signaling pathway; 31. Regulation of synaptic plasticity; 32. Negative regulation of translation; 33. Semaphorin-plexin signaling pathway; 34. Locomotory behavior.

Maternal nematode infection significantly enriched 868 PANTHER GO terms (Supplementary Datasets 4 and 5). When reanalyzed to remove redundancy and ensure semantic similarity, infection upregulated 34 REVIGO GO terms at P7, 19 of which were involved in biological processes central to postnatal mouse brain development, namely central nervous system development, 9 were in regulation of small GTPase-mediated signal transduction, and 4 were in synapse organization (Fig. 3).

#### KEGG pathway analysis

**Figure 4:**
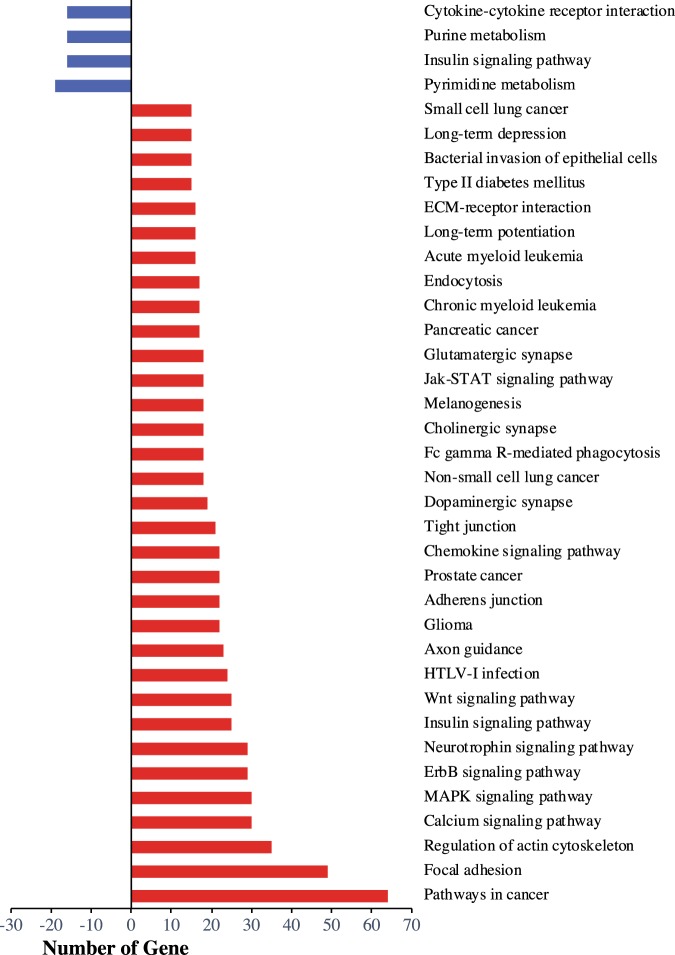
Bar diagram of KEGG pathways that were significantly down-regulated (blue) or up-regulated (red) by maternal nematode infection in the postnatal day 7 (P7) pup brain, and that had more than 15 down- or upregulated genes respectively.

Differential expression was detected for 99 pathways (Supplementary Dataset 6). Among the 37 pathways with at least 15 differentially expressed genes (Fig. 4), maternal nematode infection down-regulated three pathways: pyrimidine metabolism, purine metabolism, and cytokine-cytokine receptor interaction. The insulin signaling pathway was both down-regulated and up-regulated. Maternal infection up-regulated 30 pathways including three pathways directly involved in synaptogenesis (axon guidance, cholinergic synapse, and dopaminergic synapse) and five specifically associated with glutamatergic long-term potentiation (glutamatergic synapse, Wnt signaling, MAPK signaling, calcium signaling, and long-term potentiation).

#### Protein-protein interaction network

**Figure 5:**
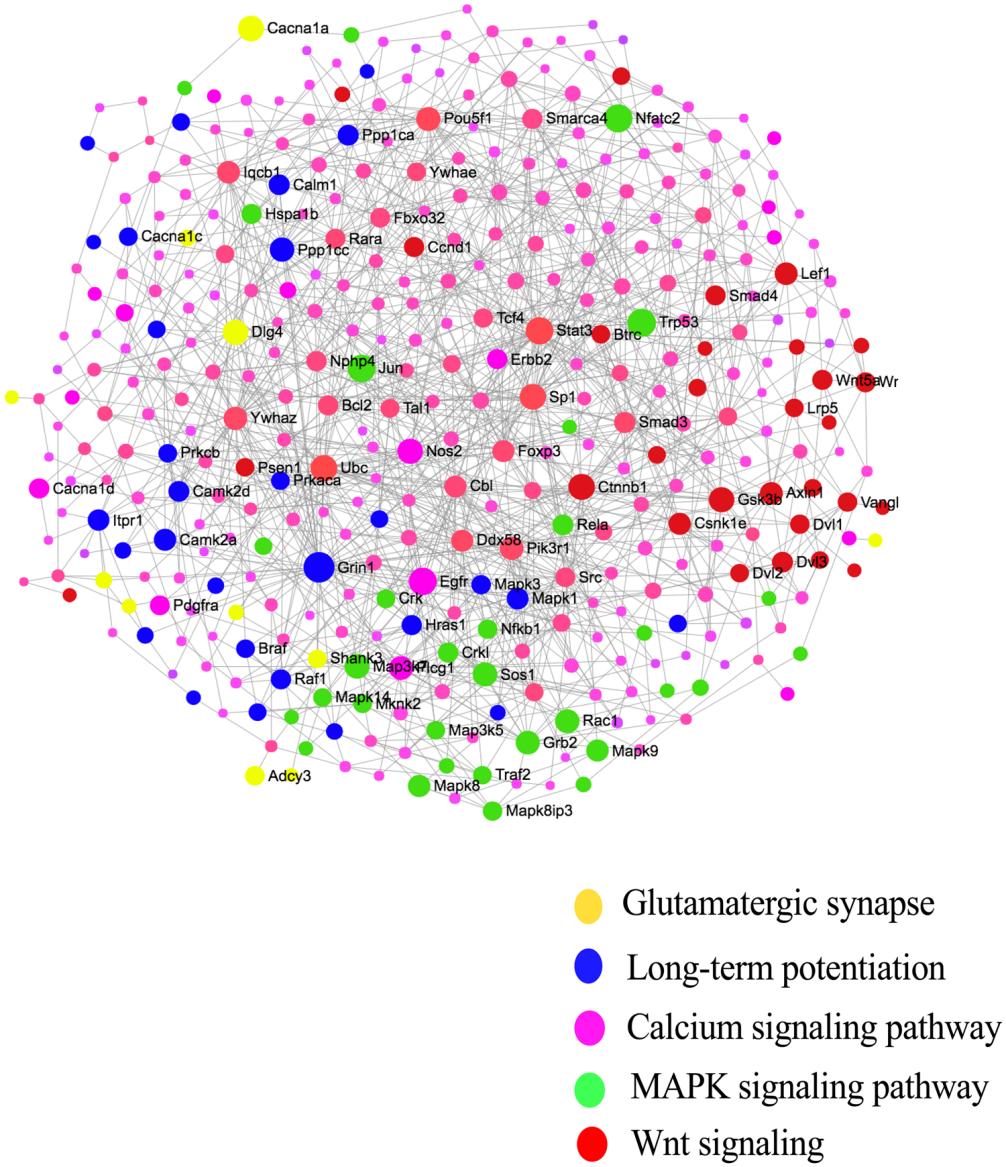
IMEX Network of the postnatal day 7 (P7) brain showing the interactions among Wnt signaling, MAPK signaling, calcium signaling, long-term potentiation and glutamatergic synapse pathways in response to maternal nematode infection.

The IMEx protein-protein interaction network confirmed that the five pathways associated with long-term potentiation interacted with one another and that maternal nematode infection altered expression of a very large number of the nodes (Fig. 5). In addition, several pathways that promote cell division and growth were up-regulated including ErbB signaling and Jak-STAT signaling which presumably indicates more favorable conditions for cellular proliferation in the pup brains of the infected compared with the control group.

### Maternal nematode infection up-regulated five pathways associated with long-term potentiation

**Figure 6:**
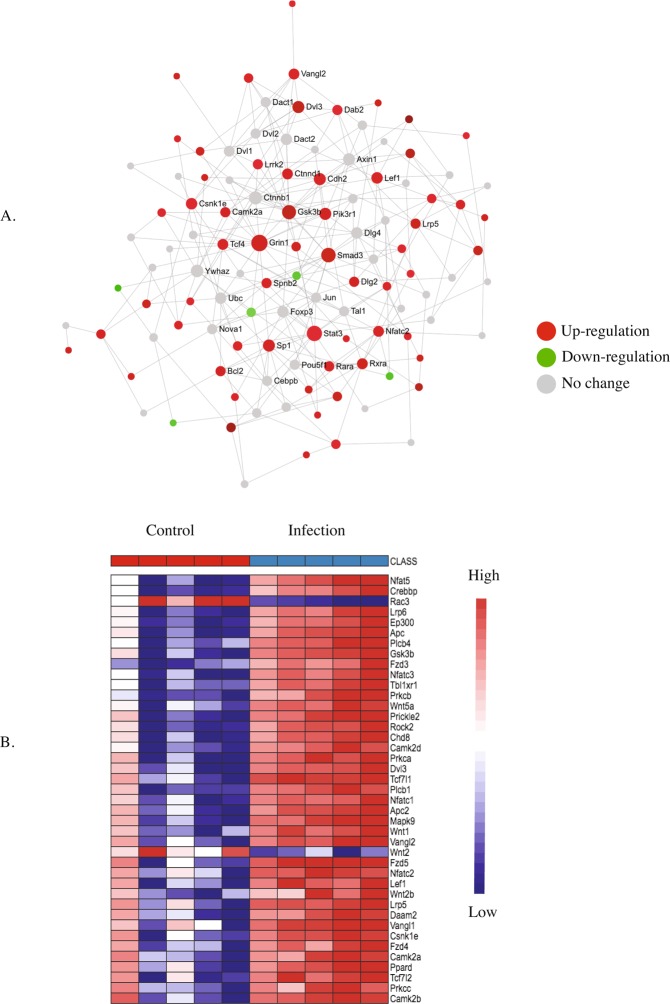
KEGG pathway analysis (**A**) and heatmap (**B**) of the postnatal day 7 (P7) brain showing up- and down-regulated genes involved in the Wnt signaling pathway in response to maternal nematode infection.

**Figure 7:**
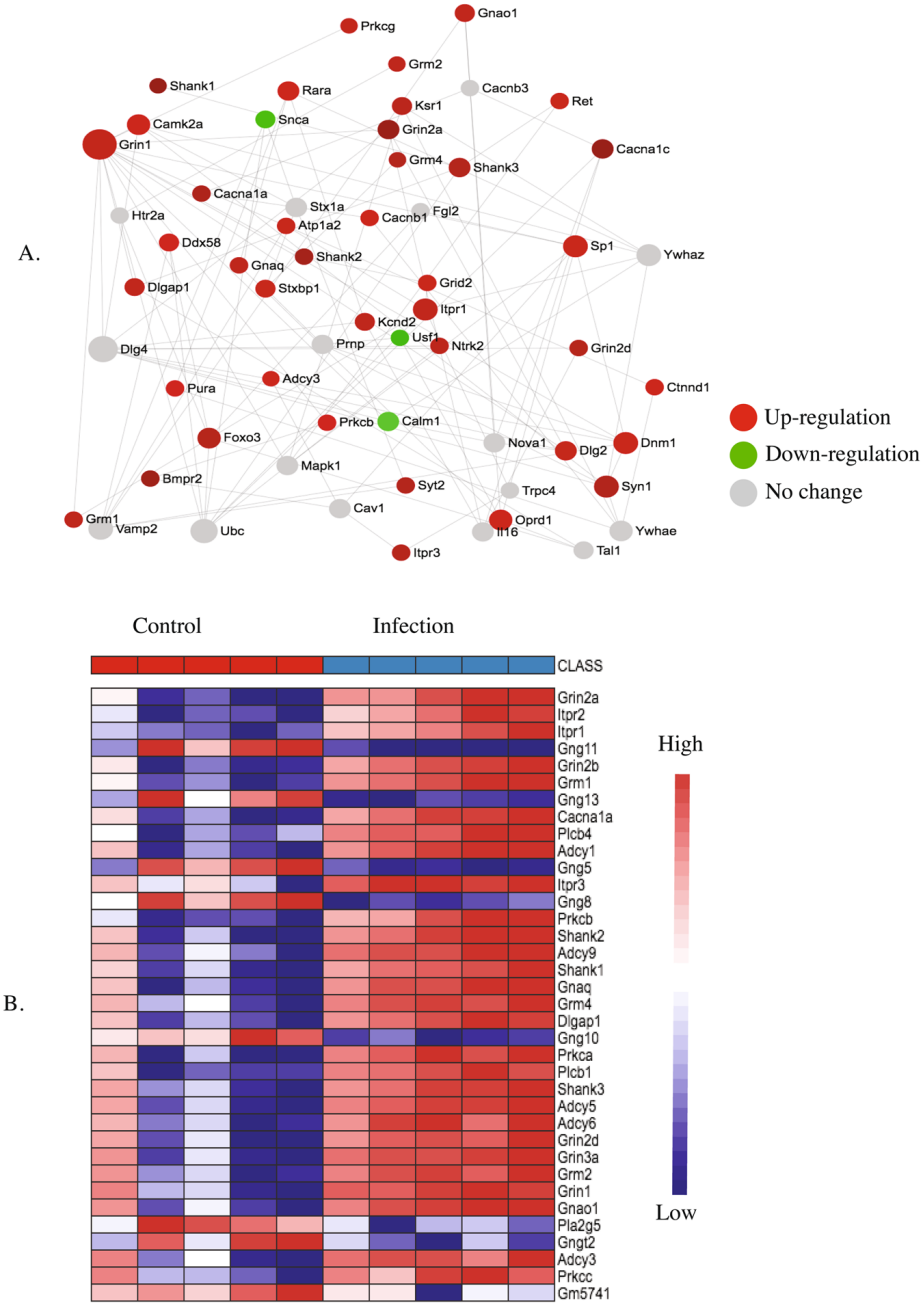
KEGG pathway analysis (**A**) and heatmap (**B**) of the postnatal day 7 (P7) brain showing up and downregulated genes involved in glutamatergic synapse signaling pathway in response to maternal nematode infection.

**Figure 8:**
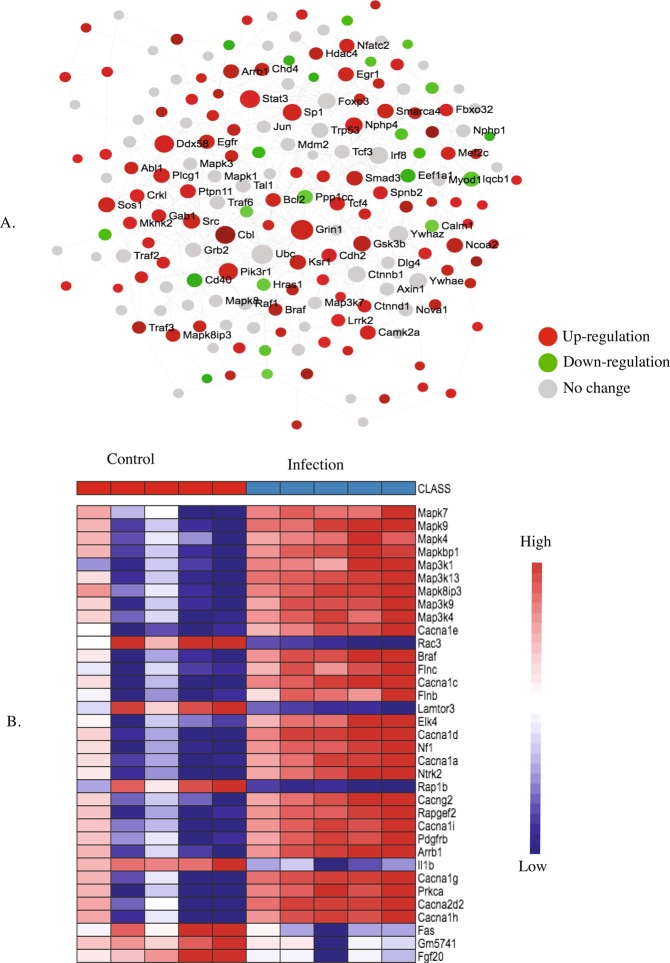
KEGG pathway analysis (**A**) and heatmap (**B**) of the postnatal day 7 (P7) brain showing up and downregulated genes involved in MAPK signaling pathway in response to maternal nematode infection.

**Figure 9:**
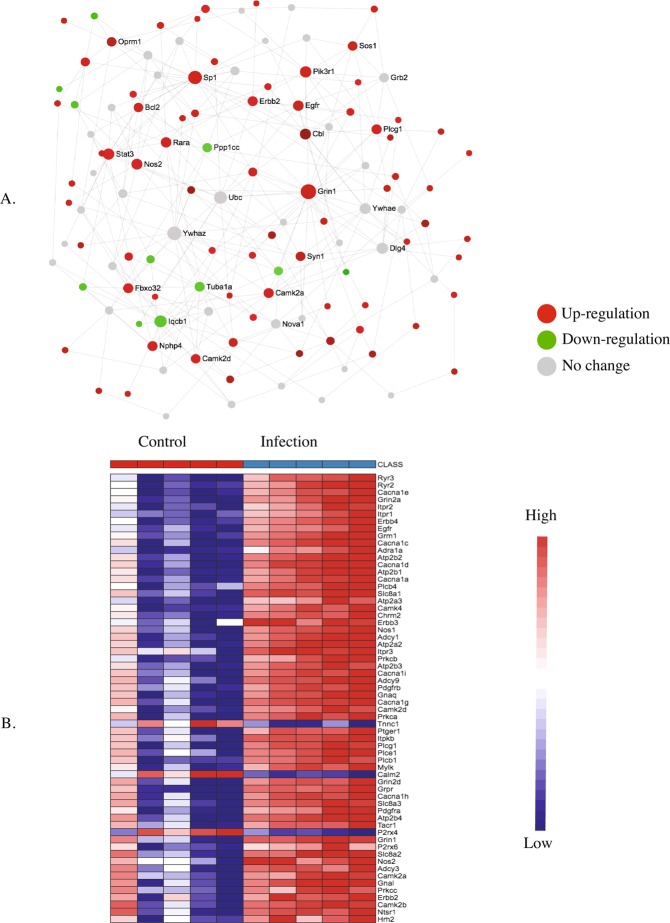
KEGG pathway analysis (**A**) and heatmaps (**B**) of the postnatal day 7 (P7) brain showing up and down-regulated genes involved in calcium signaling pathway in response to maternal nematode infection.

**Figure 10:**
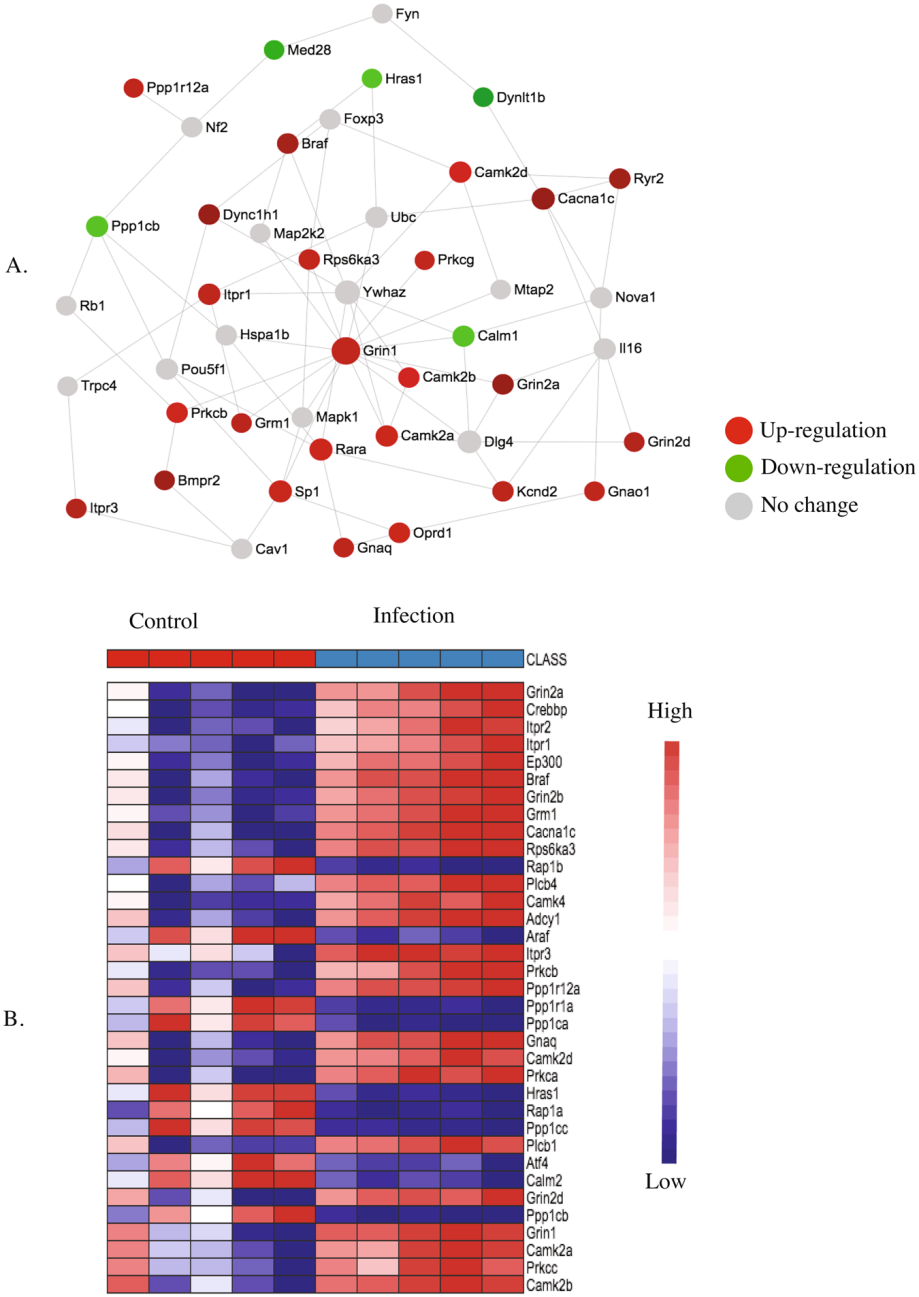
KEGG pathway analysis (**A**) and heatmap (**B**) of the postnatal day 7 (P7) brain showing up and down-regulated genes involved in long-term potentiation signaling pathway in response to maternal nematode infection.

We interrogated the pathways and heatmaps for specific critical genes involved with Wnt signalling (Fig. 6), glutamatergic signalling (Fig. 7), MAPK signalling (Fig. 8), calcium signalling (Fig. 9) and long-term potentiation (Fig. 10) at P7. In addition, the P7 database of differentially expressed genes was examined for specific genes known to be associated with long-term potentiation. The fold-change values for all differentially expressed genes at P7 is presented in the Supplemental Dataset 1.

Wnt signalling of the pre-synaptic cell is a first step in the cascade leading to long-term potentiation. Among the genes in the Wnt signalling pathway (Fig. 6), the important up-regulated genes for canonical Wnt signalling were frizzled family receptors (*Fzd*), catenin cadherin-associated proteins (*Ctnnd1* and *Ctnnd2*), dishevelled segment polarity protein 3 (*Dvl3*), glycogen synthase kinase 3 beta (*GSK3b*), and low-density lipoprotein receptor-related proteins (*LRP1, 2, 3, 4, 5*, 6, and 8). In addition, up-regulation of non-canonical Wnt signalling was evidenced by up-regulation of Wnt family members including *Wnt1*, 2*b*, and 5a, associated receptor tyrosine kinase-like orphan receptors (*RoRa* and *b*), several phospholipase Cs (*PLCs*), several potassium and calcium voltage-gated channels (*KCNQs* and *VGCC*s, respectively), lymphoid enhancer binding factor 1 (*Lef1*), transcription factor 7 like 1 and 2 (*Tcf7l 1* and *Tcf7l 2*), Creb binding protein (*Crebbp*), and several G-protein coupled receptors (*GPCR*s). Furthermore, we also recorded up-regulation of the abnormal spindle microtubule assembly (*Aspm*) gene that positively regulates Wnt signaling in the brain^22^.

The presence of excitatory glutamatergic receptors (NMDARs and AMPARs) on the post-synaptic cell is critical not only for the functioning of the glutamatergic synapse (Fig. 7), but also for long-term potentiation (Fig. 10). Two sub-units of NMDARS, *NR2A* and *NR2B* (also known as *Grin2A* and *Grin2B*) as well as several AMPARs including the metabotropic glutamate receptors 1 and 2 (*mGlu-R1* and *mGlu-R2*), *Gria3*, *Grm2*, *Grm4*, *Grik3* and *Grik5* were up-regulated in the pup brain on P7 in response to maternal nematode infection.

Up-regulation of MAPK signalling (Fig. 8) is also required for long-term potentiation (Fig. 10). Braf transforming gene (*Braf*) was up-regulated and it is involved in both MAPK signalling and long-term potentiation. Other up-regulated genes in the MAPK pathway included 11 MAPKs, guanine-nucleotide exchange factor (*Rapgef2*) and genes for several GTPase-activating proteins (*ras-GAPs)* including guanine-nucleotide exchange factors (*ras-GEF*). A number of calcium channel genes were also up-regulated including several subunits of *Cacna 1* and *Cacna 2*.

Calcium signalling (Fig. 9) is also required for MAPK (Fig. 8), glutamatergic signalling (Fig. 7) and long-term potentiation (Fig. 10). Expression of calcium/calmodulin dependent protein kinase II (*CaMK* 2) was up-regulated and it is an important mediator in the Ca^2+^ signaling pathway.

Finally, long-term potentiation is negatively associated with protein phosphatase kinases^23^. Consistent with our finding that the long-term potentiation pathway was up-regulated, expression of several protein phosphatase kinases (*Ppp1ca*, *Ppp1cc*, and *Ppp1cb*), was down-regulated in the long-term potentiation pathway (Fig. 10). Taken together, these gene expression and pathway data provide compelling evidence that long-term potentiation was up-regulated in the P7 brain of uninfected offspring of mothers that were infected with a gastrointestinal nematode throughout pregnancy and the first week of lactation.

### Maternal nematode infection up-regulated TH2 responses and down-regulated Th1 responses

Consistent with the Th2-mediated immune tolerance induced by *H. bakeri*, we observed up-regulated expression of the Th2 cytokine, IL-4, a hallmark of *H. bakeri* infection^16^ and of forkhead box protein 3 (*Foxp3*) and transforming growth factor beta (*TGFβ*) genes which exert immune tolerance effects in response to helminth infection^24^, as well as interleukin 6 signal transducer (Il6st), interleukin 6 receptor (*Il6ra*), signal transducer and activator of transcription 3 (*Stat3*), and interleukin 17 receptor (*Il17rd*) (Supplementary Dataset 1). In contrast, we observed down-regulation of several Th1-associated responses. Expression of the pro-inflammatory cytokine gene, *IL-1β*, was down-regulated, the cytokine-cytokine receptor interaction pathway was down-regulated (Supplementary Dataset 6), and several autoimmune disease pathways associated with Th1 immune activation were down-regulated (autoimmune thyroid disease, systemic lupus erythematosus, type 1 diabetes mellitus, asthma) (Supplementary Dataset 6). Together these observations showed that maternal infection was associated with up-regulation of Th2 responses and down-regulation of Th1 inflammatory responses in the postnatal (P7) brain.

## Discussion

This study extends evidence of maternal effects on early brain development by revealing changes at P7 but not P2 in pup brain gene expression in response to infection with a nematode parasite that is restricted to the maternal small intestine. Maternal nematode infection up-regulated five highly integrated pathways that are required for synaptogenesis, synaptic plasticity and long-term potentiation. Maternal infection also shifted expression of brain cytokines in favour of a Th2 response indicating an anti-inflammatory environment in the P7 pup brain. Together, these findings raise the intriguing possibility that this maternal nematode infection may have a beneficial impact on pup brain development.

Considerable neurodevelopment occurs during the perinatal period in mice. By E18 synaptic connections between neural axons and dendrons have started to form and astrocyte production increases in the first week after birth^6^. Astrocytes secrete thrombospondin (TSPs) proteins which are crucial for synaptogenesis^19^. Under normal conditions, most neurons have established synaptic connections forming neural circuits by P7^6^. This early brain development is controlled by precise expression of genes related to transcription factors, receptors, ion channels, synaptic proteins, cell adhesion molecules, cell signaling pathways and other associated factors^6^ with higher expression at P7 compared to E18^6^. Our results extend these findings in that maternal infection further increased expression of these genes at P7. Moreover, our observation of up-regulation of essential genes for brain development including *Tsp1*^19^, *Otx1*^20^ and *Bptf*^21^ suggests that maternal nematode infection may have a positive impact on the developmental progression of gene expression from E18 to P7.

Glutamatergic synaptic receptors are a type of excitatory receptor in the brain that play a crucial role in neuronal development, synaptic plasticity and long-term potentiation^25^. NMDARs are one of the main ionotropic glutamatergic receptors and consist of NR2A and NR2B subunits whose differential expression pattern depends on the stage of brain developmental^26^. In mice, expression of *NR2B* is up-regulated from birth and throughout the postnatal development whereas expression of *NR2A* begins around P7 and increases until 2 or 3 weeks before adulthood^27^. In response to maternal nematode infection, expression of both *NR2A* and *NR2B* (also known as *Grin2A* and *Grin2B*) genes was higher in the P7 brain. Such early up-regulation of NMDAR sub-unit expression in response to prenatal exposure to valproic acid was shown to result in synaptic plasticity and long-term potentiation in neocortical pyramidal neurons^28^ whereas lower NMDAR expression in response to maternal stress resulted in learning deficits in offspring^29^. Based on our findings, pups of dams infected with an intestinal nematode during pregnancy and early lactation may have improved NMDAR-mediated synaptic plasticity and long-term potentiation.

Ca^2+^ signalling is required for NMDAR-mediated synaptic plasticity and long-term potentiation^30^, and the Ca^2+^ signalling pathway was up-regulated in pups of nematode-infected dams. The activation of NMDARs accelerates the intracellular influx of Ca^2+^ ions which activate CaMK*2* which subsequently auto-phosphorylates the synaptic AMPARs, enhancing the conductance of the receptor leading to long-term potentiation^31,32^. Furthermore, Ca^2+^ signaling activates the transcription factor cAMP response element binding protein (*Crebbp*) which enhances transcription of genes involved in neural growth and survival as well as synaptic connectivity^33^. The higher expression of both *CaMK2* and *Crebbp* as well as the Ca^2+^signaling pathway itself in the brain of P7 pups of nematode-infected dams indicates up-regulated Ca^2+^ signalling, and furthermore, down-regulation of protein phosphatase kinases which suppress long-term memory^23^ supports the observation that maternal nematode infection exerts a positive influence on the developing brain of the pups.

Maternal nematode infection also up-regulated the Braf-MAPK signaling pathway that is essential for synaptic transmission and long-term potentiation^34^. Braf-MAPK signalling provides the phosphorylation link between activated CaMK2 receptors and AMPARs^35^. Small GTPases initiate a phosphorylation reaction cascade through *ras-GEF* and *ras-GAP* that were both up-regulated. Phosphorylation of AMPARs, including the up-regulated metabotropic glutamate receptors 1 and 2 (*mGlu-R1* and *mGlu-R2*), leads to their expression on the post-synaptic membrane resulting in synaptic plasticity and long-term potentiation^36^. Deficiency of *mGlu-R1* has been shown to impair motor function and synaptic plasticity^37^. Hence, up-regulation of the Braf-MAPK pathway together with enhanced expression of key genes including the Braf transforming gene (*Braf*), several *MAPK*s, *Ras-GEF*, and *Ras-GAP* in the pup brain would also contribute to synaptic plasticity and long-term potentiation.

Wnt signaling is needed for transcription of genes involved in synaptic plasticity and long-term potentiation^38^, and this pathway was up-regulated in our study. Canonical Wnt signalling through β-catenin mediates neural differentiation from the neural precursor cells^39^. Several genes associated with canonical Wnt signaling were up-regulated in the P7 brain of nematode-infected dams including *Fzd3, catenin* and several *LRPs*. Non-canonical Wnt signalling through Ca^2+ ^^40^ leads to higher expression of NMDARs in the synapses, release of intracellular Ca^2+^ *via* voltage-gated Ca^2+^ channels, and synaptic responses including long-term potentiation^3^. We also have evidence for up-regulation of non-canonical Wnt signalling as seen in higher pup brain expression of *Wnt5a*, its associated receptors (*RoRa* and *b, PLC*) and ion channels (*KCNQs* and *VGCC*) in response to maternal infection. Our findings differ from the observation that maternal exposure to toxic chemicals down-regulates non-canonical Wnt signaling and impairs growth of a range of tissues in offspring^41,42^. Both higher canonical and non-canonical Wnt signaling observed in our study further strengthen our conclusion that *in utero* and postpartum exposure to a nematode restricted to the maternal intestine may improve synaptic plasticity in the next generation.

The exact mechanism whereby a nematode living in the lumen of the maternal intestine would augment the signalling pathways associated with synaptic plasticity and long-term potentiation in the P7 pup brain is not clear, but our data raise two possibilities. First, maternal nematode infection reduced neuro-inflammation in the pup brain as evidenced by the down-regulated expression of the inflammatory cytokine *IL-1*β which impairs neurogenesis and cognition^43^ and up-regulated expression of the anti-inflammatory cytokine *IL-4* which has been previously shown to promote long-term potentiation^44^. Moreover, up-regulation of *Foxp3*, *TGF*β, *Il6st*, *Il6ra, Stat3* and *Il17rd* strongly suggest that maternal nematode infection induced an immune tolerant or anti-inflammatory environment which is known to promote long-term potentiation in the pup brain^45^. *IL17* has also been shown to have anti-inflammatory properties and to suppress the development of autoimmune disease^46^. Thus, the up-regulation of *IL17* expression in response to maternal infection is consistent with our observation that autoimmune-related pathways (autoimmune thyroid disease, systemic lupus erythematosus, type I diabetes mellitus) and immune activation related (asthma) disease pathways in the pup brains were down-regulated in response to maternal infection. Second, it is possible that *H. bakeri* derived microRNA^47^ might reach the neonatal circulation and influence Wnt signaling in the postnatal brain. We recorded up-regulated expression of *Aspm* gene which interacts with small interfering RNA^48^ and is a positive regulator of Wnt signaling in the brain^22^.

Previously, a few beneficial effects of nematode parasites have been demonstrated in humans including use of nematode eggs to treat autoimmune diseases^49^, lower allergic responses in nematode-infected infants and children^50^ and favourable effects of nematodes on maternal reproduction as evidenced by improved conception, implantation and overall fecundity^51^. Regarding cognition, although there is some evidence from school-age children that direct infection with intestinal nematodes impairs cognition^52,53^, a meta-analysis of the human studies concludes that the body of evidence does not support this claim^54^. In rodent study, an intestinal nematode infection with an extra-intestinal phase has been shown to impair reference memory in adult mice^55^. However, prior to the present study, nothing was known about the impact of the maternal nematode infection directly on postnatal brain gene expression or more specifically on key neuro-developmental pathways, although two epidemiological studies are of relevance. Both have identified a negative, not positive, association between maternal infection with two intestinal nematodes during pregnancy and cognitive scores in infants^56,57^. Of note, however, both of the human nematodes have an extra-intestinal phase^58^, in contrast to our model where the nematode remains in the intestine^16^. It is likely that researchers did not know whether the mothers had been infected during pregnancy, and that maternal infection during pregnancy and lactation may have heightened long-term potentiation with long-lasting beneficial effects for their children. Of course, this possibility can only be entertained if the rodent-based findings extend to humans.

In conclusion, our study demonstrated that a gastrointestinal nematode infection restricted to the maternal intestine positively influenced expression of postnatal brain genes and pathways associated with long-term potentiation. To the best of our knowledge, this is the first study showing that maternal nematode infection influences postnatal brain gene expression. This sheds light on an unappreciated effect of maternal nematode infection on postnatal brain development, which may have important long-term consequences for learning and cognition. However, further research is needed to determine whether differential gene expression leads to altered neurophysiology and altered cognitive behaviors that persist as the pups grow. If also applicable to human populations, this finding may have relevance to decisions regarding deworming treatments during pregnancy and early lactation.

## Materials and Methods

### Experimental design

We used a randomized design where half of the pregnant mice were infected with an intestinal nematode, and half were given a sham infection. Litters were sacrificed either on postpartum day 2 (P2) and postpartum day 7(P7). The protocol (#2000–4601) was approved by the McGill University Animal Care Committee according to the guidelines of the Canadian Council on Animal Care.

### Parasites and mice

*Heligmosomoides bakeri* (=*H. polygyrus*; *Nematospiroides dubius*) is a murine nematode model of intestinal nematodes of livestock and humans^16^. When ingested, infective third stage larvae (L_3_) penetrate the submucosa of the small intestine and undergo two moults before returning to the intestinal lumen where adult worms release eggs in the faeces^16^. Repeated exposure to larvae stimulates an ongoing Th2 immune response^18^.

**Figure 11:**
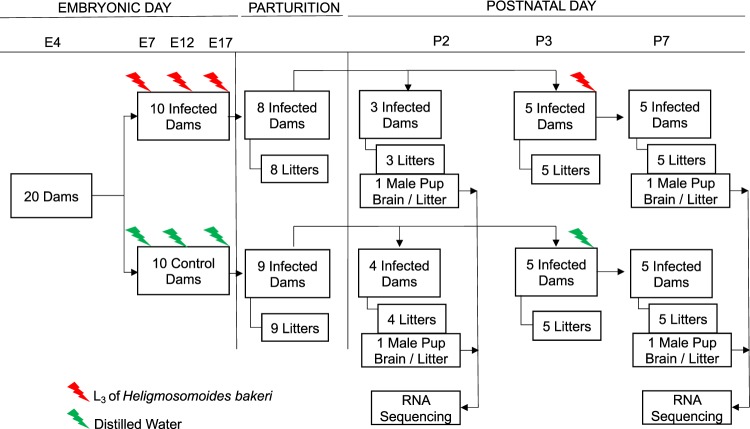
Schematic representing experimental design. One male pup brain per litter was collected for RNA sequencing at postnatal (P) days 2 and 7. Although 20 timed-pregnant dams were received and allocated to treatments on embryonic day (E) 4, only 17 delivered litters at parturition.

A total of 20 primiparous 8 to 9-week-old timed pregnant (gestation day, E4) outbred CD1 mice were housed individually in Nalgene cages (Fisher Scientific, Canada) with stainless steel racks to prevent coprophagia, at 22–25 °C, 40–60% relative humidity and a 12 h light and dark cycle. Mice were fed a 24% protein diet (Harlan Teklad, TD. 90017) *ad libitum*. On embryonic day 5 (E5), mice were separated randomly into two groups (control and infected). The infected group was intubated with 100 ± 3 (L_3_) suspended in distilled water on E7, E12 and E17, as well as on P3, for those mice necropsied on P7 (Fig. 11). Establishment of *H. bakeri* infection in infected dams was confirmed at necropsy by presence of lesions on the duodenal wall and adult worms in the intestinal lumen. The control group was intubated with the same volume of distilled water on all infection days.

### Pup brain and liver collection

We did not obtain litters from one control and two infected dams on P2. Therefore, four litters from control and three litters from infection group were selected and exsanguination on P2. Similarly, on P7, the remaining five litters from each group were sacrificed and the whole brain and liver tissue were collected. All tissue samples were snap frozen in liquid nitrogen and stored at −80^0^C.

### Genetic determination of sex of pups from liver tissue

Genomic DNA was extracted from pup liver tissue by DNeasy Blood & Tissue Kit® (Qiagen, Germany) following the manufacturer’s guideline and amplified using the SX_Forward, 5′-GATGATTTGAGTGGAAATGTGAGGTA-3′ and SX_Reverse, 5′-CTTATGTTTATAGGCATGCACCATGTA-3′ primer pair according to published protocol^59^. Polymerase chain reactions were performed in a final volume of 25 µl using GoTaq® Green Master Mix (Promega, USA) with the following protocol: initial denaturation at 95 °C for 5 min, 30 cycles with 95 °C for 1 min, 85 °C for 30 secs, 75 °C for 30 secs, 53 °C for 1 min, 72 °C for 1 min. and the final elongation at 72 °C for 5 min. The amplified products were run on 2% agarose gel with 2-Log DNA Ladder (0.1–10.0 kb, New England BioLabs). The primers amplified a Y-chromosome specific fragment in males that produced a 280 bp amplicon and two fragments of X-chromosome in females producing 480 and 660 bp amplicons.

### RNA extraction and Illumina Hi-Seq sequencing of the male pup brain

For this study, we only studied the brains of male pups as the hippocampus is larger in males than females in early postpartum^60^. At both P2 and P7 time points, one brain per litter was randomly selected, and the total RNA of each brain was extracted using a commercial kit (NucleoSpin^®^ RNA Plus, Macherey-Nagel, Germany) following the manufacturer’s protocol. No pooled samples were used. The concentration of RNA was determined spectrophotometrically using NanoDrop. Seven RNA samples from P2 (four from the control and three from the infection group) and 10 RNA samples from P7 (five from the control and five from the infection group) were sent to Genome Quebec, McGill University for cDNA library preparation and Illumina Hi-Seq sequencing using the Illumina HiSeq. 4000 sequencer. After initial processing of raw data (FASTQ) including filtering of low tags, trimming, adaptor removal and alignment to the mouse reference genome (GRCm38), the sequencing centre provided us with the paired-end BAM files. The data files have been submitted into the Gene Expression Omnibus (GEO) repository (GSE118064).

### Counting reads and analysis of differential gene expression

The paired end BAM files were sorted by read name using SAM tools^61^. HTSeq-count (v0.6) was used to count the reads for expressed exons within each gene^62^. All the count tables from different BAM files were compiled categorically (infection *vs* control) in a tab delimited text (.txt) file for each time point (P2 and P7) and the text files were uploaded to *NetworkAnalyst* (http://www.networkanalyst.ca), a web-based tool for comprehensive gene expression profiling^63^. The *edgeR* method was selected with adjusted *P* value < 0.05 and fold change > 1 to identify genes differentially expressed in the pup brain in response to maternal nematode infection. Principal component analysis confirmed that the effect of maternal nematode infection was homogeneous among the experimental replicates (Supplementary Fig. 3).

### Time-series analysis of gene expression

For the time-series gene expression analysis, we used gene expression data of three time points, namely E18, P2 and P7. The E18 data were obtained from our previous experiment^14^ using the same experimental system (GSE96103) where pregnant mice had been given a sham infection or infected with 100 ± 3 L_3_ on E5, E10 and E15 (vs. E7, E12, E17 and P3 in the present study), and where brains of unsexed fetuses (vs. only male pups in the present study) were harvested on E18. The previous study had included both protein-sufficient and protein-deficient dams, but fetal brain gene expression data only from the protein-sufficient arm of the study were used here.

We used *MetaboAnalyst* 4.0 pipeline with time series and one experimental factor as study design^64^. A heatmap was created for the direct visualization of the relative levels of gene expression over E18, P2 and P7 time points and between the control and infection groups and further modified by elimination of non-significant genes by ANOVA2 with Bonferroni multiple testing. Multivariate Empirical Bayes time-series analysis (MEBA)^65^ was used in the pipeline to rank the genes whose temporal expression patterns differed. The 100 most highly ranked genes were selected and analyzed for gene ontology biological processes.

### Gene ontology enrichment analysis of differentially expressed genes

The differentially expressed genes were analysed for enrichment with Gene Ontology biological process by PANTHER overrepresentation test. The significance of enriched GO terms was determined after Bonferroni correction for multiple testing. Furthermore, the enriched GO terms were summarized by removing redundancy and clustering based on their semantic similarity using ReviGO^66^.

### Pathway analysis and interactive network visualization

Protein-protein interaction networks of the differentially expressed genes were generated using the IMEx interactome database and the biological significance of up- and down-regulated nodes was determined by functional exploration against the KEGG pathway database in *NetworkAnalyst*.

## Supplementary information

Supplementary Figures

List of differentially expressed genes at P7

Top 100 genes from time-series analysis with Hotelling-T2 value

Up-regulation and down-regulation of genes important for brain development at P7

Up-regulated gene ontology biological process terms at P7

Down-regulated gene ontology biological process terms at P7

List of KEGG pathways up and down regulated at P7
