# Supplementary material for: Maternal Gastrointestinal Nematode Infection Up-regulates Expression of Genes Associated with Long-Term Potentiation in Perinatal Brains of Uninfected Developing Pups
Source: Sci Rep. 2019 Mar 12;9:4165. doi: 10.1038/s41598-019-40729-w (PMC6414690; doi:10.1038/s41598-019-40729-w)
Supplement: Supplementary file 1 — Supplementary Figures [file 41598_2019_40729_MOESM1_ESM.pdf]

Maternal Gastrointestinal Nematode Infection Up-regulates Expression of Genes Associated with Long-Term Potentiation in Perinatal Brains of Uninfected Developing Pups

Manjurul Haque, Kristine G. Koski, Marilyn E. Scott*

Supplementary Fig. 1

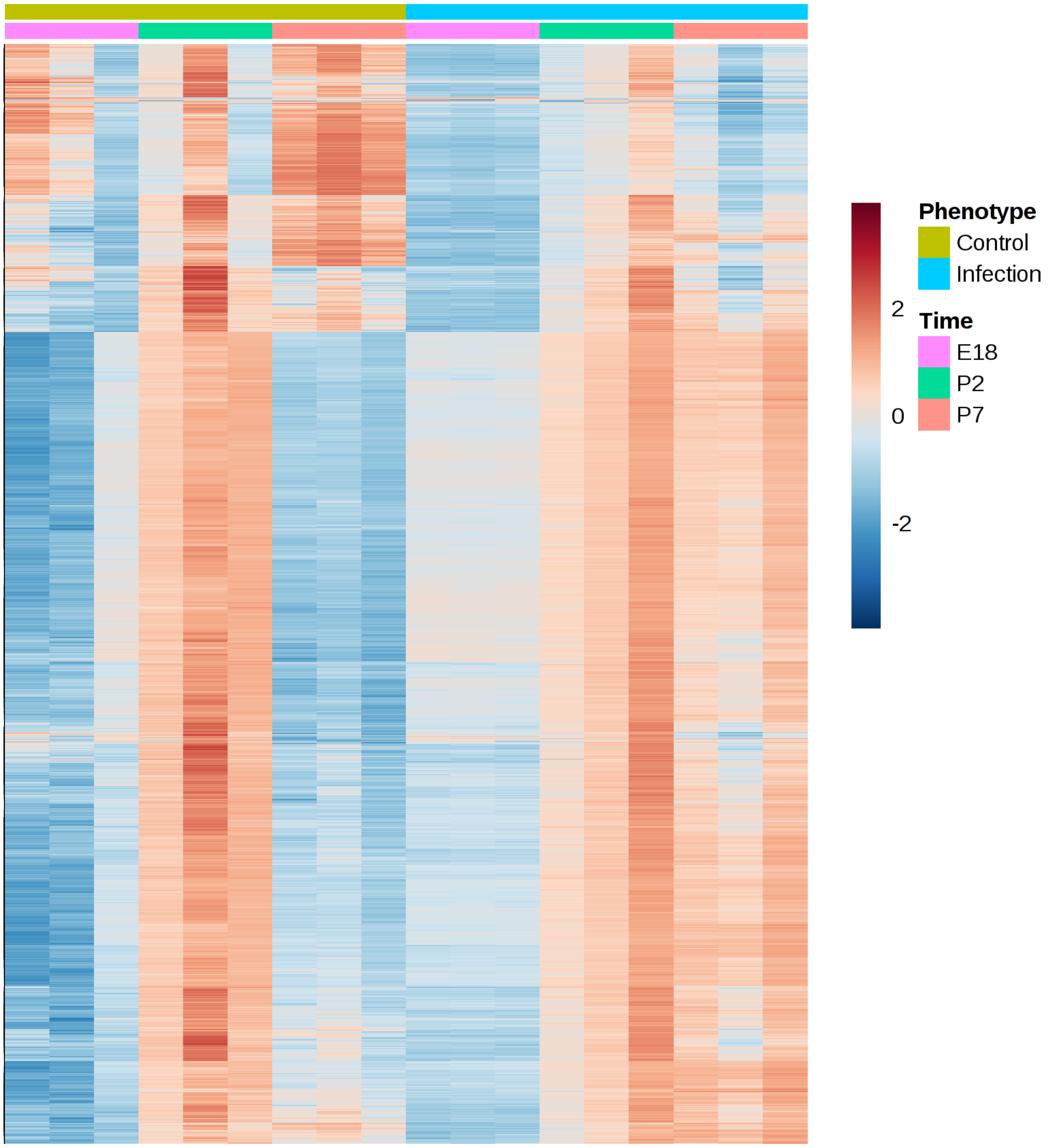

Heatmap showing the expression of all genes between control and infection groups over three time points, E18, P2 and P7.

Supplementary Fig. 2

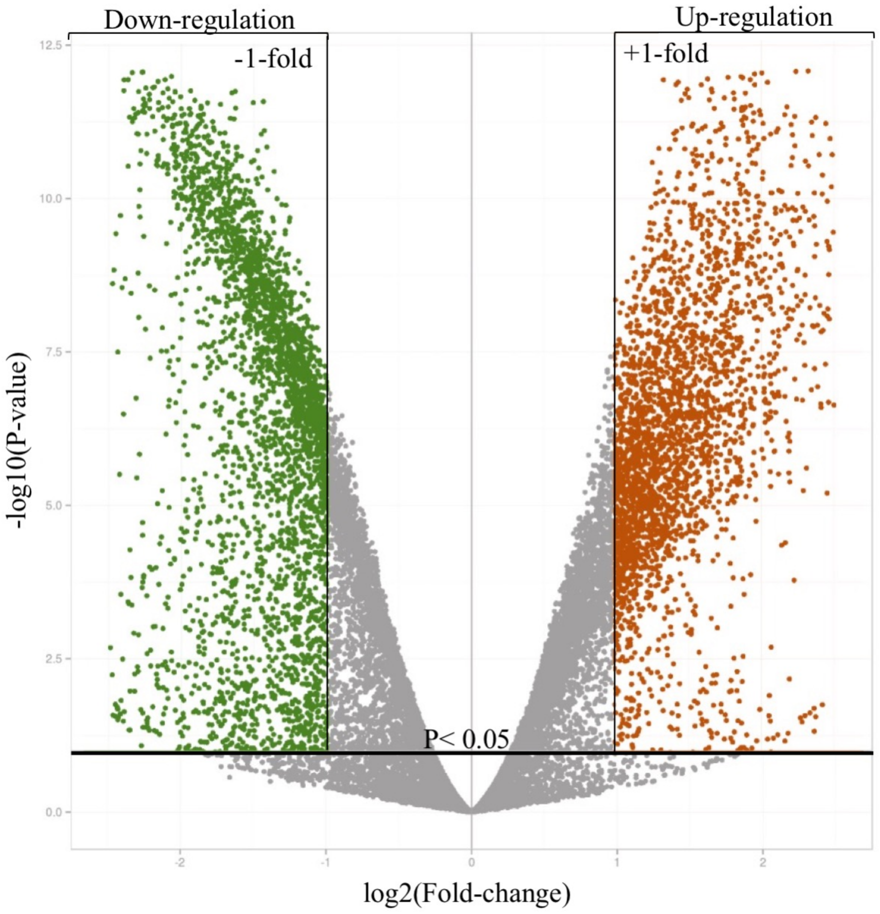

Volcano plot showing differentially expressed genes in postnatal day 7 pup brain in response to maternal nematode infection. The negative log_{10} transformed *p* values test the null hypothesis of no difference in expression levels between control and infected pups (*y* axis) and are plotted against the average log_2 fold changes in expression (*x* axis). The up-regulated genes are plotted in brown and the down-regulated genes are plotted in green (*p* value <0.05) with an absolute log_2 fold change less than or equal to 1 or greater than 1, respectively.

Supplementary Fig. 3

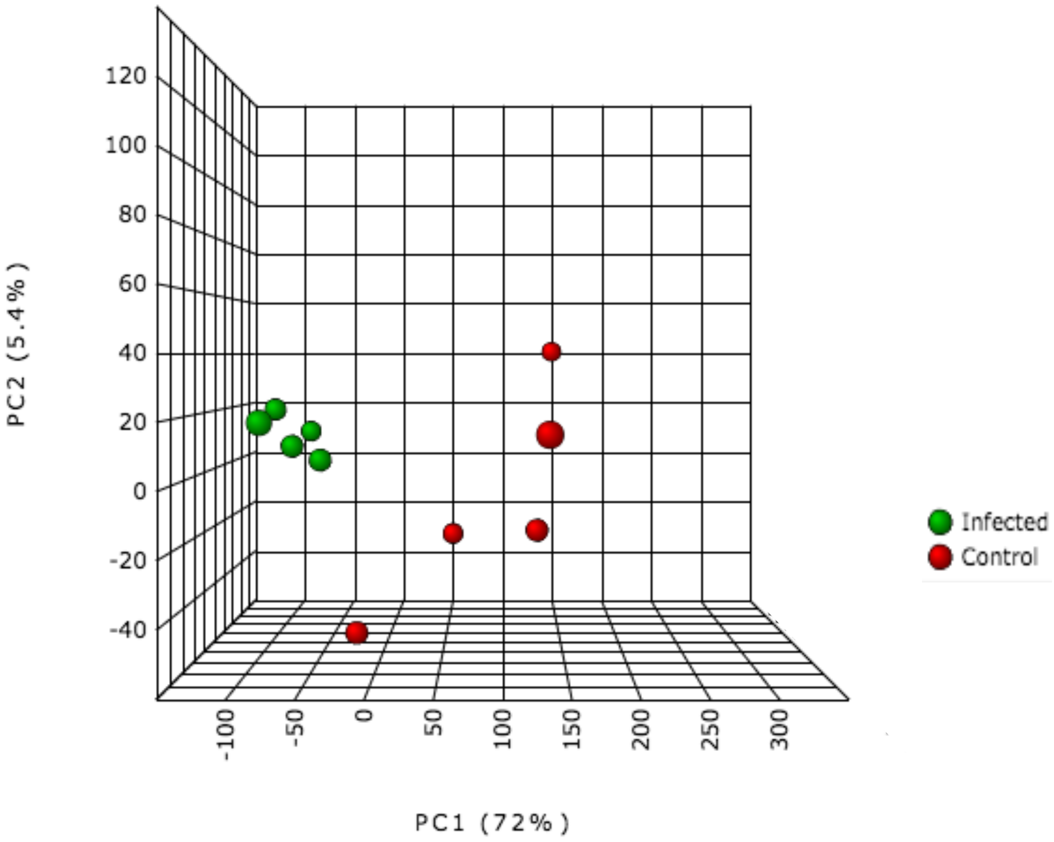

Principal component analysis showing clustering of maternal nematode infection replicates (green dots), indicating homogeneous effect of maternal nematode infection on the postnatal day 7 brain gene expression.
